# Free-breathing 3D late gadolinium enhancement imaging of the left ventricle using a stack of spirals at 3T

**DOI:** 10.1002/jmri.24643

**Published:** 2014-05-03

**Authors:** Iain T Pierce, Jennifer Keegan, Peter Drivas, Peter D Gatehouse, David N Firmin

**Affiliations:** 1 Cardiovascular BRU, Royal Brompton HospitalLondon, UK; 2 National Heart and Lung Institute, Imperial College LondonLondon, UK

**Keywords:** late gadolinium enhancement, 3D, spiral, stack of spirals, free-breathing

## Abstract

**Purpose:**

To develop navigator-gated free-breathing 3D spiral late gadolinium enhancement (LGE) imaging of the left ventricle at 3T and compare it with conventional breath-hold 2D Cartesian imaging.

**Materials and Methods:**

Equivalent slices from 3D spiral and multislice 2D Cartesian acquisitions were compared in 15 subjects in terms of image quality (1, nondiagnostic to 5, excellent), sharpness (1–3), and presence of artifacts (0–2). Blood signal-to-noise ratio (SNR), blood/myocardium contrast-to-noise ratio (CNR), and quantitative sharpness were also compared.

**Results:**

All 3D spiral scans were completed faster than an equivalent 2D Cartesian short-axis stack (85 vs. 230 sec, *P* < 0.001). Image quality was significantly higher for 2D Cartesian images than 3D spiral images (3.7 ± 0.87 vs. 3.4 ± 1.05, *P* = 0.03) but not for mid or apical slices specifically. There were no significant differences in qualitative and quantitative sharpness (95% confidence interval [CI]: 1.91 ± 0.67 vs. 1.93 ± 0.69, *P* = 0.83 and 95% CI: 0.41 ± 0.07 vs. 0.40 ± 0.09, *P* = 0.25, respectively), artifact scores (95% CI: 0.16 ± 0.37 vs. 0.40 ± 0.58, *P* = 0.16), SNR (95% CI: 121.5 ± 55.3 vs. 136.4 ± 77.9, *P* = 0.13), and CNR (95% CI: 101.6 ± 48.4 vs. 102.7 ± 61.8, *P* = 0.98). Similar enhancement ratios (0.65 vs. 0.62) and volumes (13.8 vs. 14.1cm^3^) were measured from scar regions of three patients.

**Conclusio:**

Navigator-gated 3D spiral LGE imaging can be performed in significantly and substantially shorter acquisition durations, although with some reduced image quality, than multiple breath-hold 2D Cartesian imaging while providing higher resolution and contiguous coverage..

LATE GADOLINIUM ENHANCEMENT (LGE) imaging is the current gold standard and most commonly used technique for evaluating the viability of the myocardium 1–3. The LGE imaging technique relies on the differing contrast agent kinetics between viable and nonviable tissue to provide image contrast, with scarred or fibrous tissue having an increased volume of contrast agent while also retaining the contrast agent for longer. T_1_-weighted imaging, using an inversion pulse and imaging when the longitudinal magnetization of normal myocardium is passing through the null point, results in image contrast between viable and nonviable tissue 1,2,4.

Clinical assessment of myocardial viability is generally performed using a nonselective inversion-prepared segmented gradient echo sequence with Cartesian *k*-space coverage and predominantly consists of acquiring a stack of breath-hold 2D short axis slices through the long axis of the left ventricle (LV) 5. Due to incomplete T_1_ magnetization recovery over a single RR interval, alternate R-wave gating is used to minimize sequence sensitivity to RR interval variations and to maximize magnetization recovery between data segments. Alternate R-wave gating and the need to complete each acquisition within a comfortable breath-hold period of around 10–12 seconds limits spatial resolution to ∼1.4 × 1.9 mm with imaging performed in the mid-diastolic rest period to minimize motion blurring over the acquisition windows of 145–200 msec 3.

This technique has been extended to 3D imaging allowing the short-axis stack to be acquired in a single breath-hold. This avoids the misregistration that can occur between repeated breath-hold 2D imaging due to variability in breath-hold position and also reduces patient fatigue, which may result in poor breath-holding and ghosting. In comparison to 2D LGE imaging, similar or improved spatial resolution has been attained over longer breath-holds (20–40 sec). This has been achieved by using novel sampling schemes 6,7 and/or parallel imaging 8,9 (with both partial Fourier and Fourier interpolation). Acquisition windows, however, are generally increased (120–300 msec) 6,8–10 and to complete the acquisitions within a single breath-hold period, data segments are acquired on consecutive (rather than alternate) cardiac cycles, increasing sensitivity to RR interval variations. The use of multiple smaller 3D volumes has also been used to reduce the long breath-hold durations 11, achieving up to 3 mm through-plane resolution 12.

The application of navigator gating to 3D LGE scans has enabled improvement of the through-plane resolution for imaging of the LV short-axis stack compared to 3D breath-hold methods, without the need for extended breath-holding or longer acquisition windows. Increased spatial resolution, generally through-plane, is achieved at the expense of increased total scan duration. As with 3D breath-hold imaging, partial Fourier and interpolation are common, with single R-wave gating used to reduce scan durations to acceptable levels. Free-breathing 3D LGE imaging provides similar image quality to 3D breath-hold LGE imaging 13 and improved image quality, signal-to-noise ratio (SNR), and contrast-to-noise ratio (CNR) compared with breath-hold 2D 14–16. However, while typical respiratory efficiency for navigator scans is 40–50%, irregular breathing patterns and respiratory drift are common and can result in highly variable and unpredictable acquisition durations. In addition, residual chest wall motion may result in respiratory artifacts such as chest wall ghosting, and optimal inversion time selection can change throughout the longer scan durations due to gadolinium washout. Free-breathing 3D LGE imaging has also been performed to assess left atrial scar following RF ablation of atrial fibrillation 17,18. For this application the thin atrial wall requires higher spatial resolution imaging which extends acquisition durations further.

The majority of LGE imaging has been performed at field strengths of 1.5T. Imaging at 3T should result in increased SNR. The CNR should also be increased as the T_1_ of normal myocardium is longer at higher field strength. While this has been observed in some studies 19, others have shown no such increase 20. Single R wave LGE imaging at 3T may be more problematic, shown by comparisons of alternate R-wave gated 2D imaging and single R-wave gated 3D imaging at 3T 12,16.

Spiral trajectory readouts have highly efficient *k*-space coverage per excitation 21 and can be applied as a stack of spirals 3D acquisition using conventional through-plane phase encoding 22. The increased efficiency of spiral imaging has the potential to improve the spatial resolution without significantly prolonging scan time, as recently demonstrated for atrial imaging at 1.5T 23. Alternatively, the total acquisition duration can be much reduced with acceleration techniques 24 to allow full coverage of the LV in a single breath.

In this study we aimed to use the increased efficiency of spiral imaging to develop a free-breathing, navigator-gated 3D spiral LGE sequence for imaging the LV in typically under 2 minutes. Unlike the previous stack of spirals studies 23,25, this technique was developed at 3T where off-resonance effects are more problematic. We propose that this technique will allow faster coverage of the LV than conventional breath-hold 2D Cartesian imaging without compromising image quality.

## MATERIALS AND METHODS

In all, 18 consecutive patients, 10 male, mean age 53 years (range 33–83), undergoing conventional LGE studies for a range of indications: assessment of oncology patients (nine patients), cardiomyopathies (hypertrophic cardiomyopathy (HCM) three patients, dilated cardiomyopathy (DCM) two patients, arrhythmogenic right ventricular cardiomyopathy (ARVC) 1 patient), myocarditis (one patient), and sarcoidosis (two patients) were recruited under approved local ethics providing informed written consent. For all patients, conventional breath-hold 2D Cartesian LGE scanning commenced ∼8 5–15 minutes after contrast agent (0.1 mmol/kg gadobutrol [Gadovist]) was given with the inversion time altered to null the myocardium for each scan. All conventional breath-hold 2D LGE imaging was completed prior to free-breathing 3D spiral LGE imaging so as not to compromise the clinical study. The free-breathing 3D spiral imaging was started ∼18 11–24 minutes after contrast agent administration. The durations of the series of breath-hold 2D Cartesian scans (including rest periods between breath-holds) and of the free-breathing 3D spiral scans were recorded and compared using a paired Wilcoxon signed rank sum.

Scans were performed on a Siemens (Erlangen, Germany) 3T Skyra MR Scanner with an 18 channel body matrix surface coil and appropriate spine coils embedded in the scanner couch.

### Breath-Hold 2D Cartesian

Conventional breath-hold 2D Cartesian imaging was performed using an inversion-prepared segmented fast gradient echo sequence (TE = 1.6 msec, TR = 4.1 msec, flip angle = 20°). The frequency encoding field of view was 350 to 380 mm, with the phase encoding field of view adjusted on a patient basis. The acquired resolution was 1.4 × 1.8 mm and interpolated to 0.7 × 0.7 mm during reconstruction. The slice thickness was 7 mm with a slice gap of 3 mm, requiring 8–12 short-axis slices for full coverage of the LV from outflow tract to apex. Acquisition of 32–52 data lines per segment resulted in acquisition windows of 141–210 msec. Data were acquired over 8–16 cardiac cycles (depending on breath-hold capability) with gating on alternate R waves.

### Free-Breathing 3D Spiral

**Figure 1:**
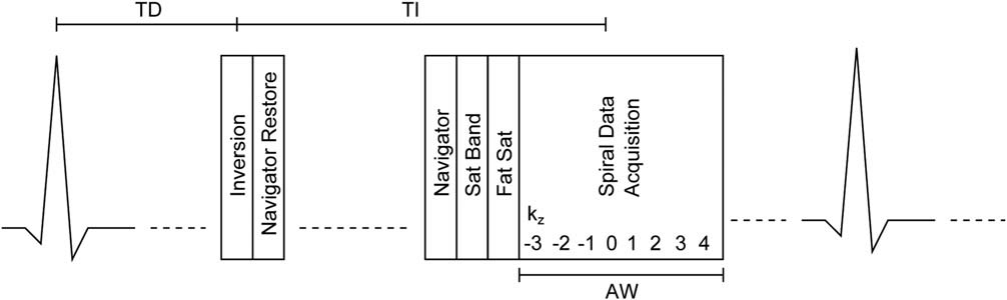
Sequence timing of the navigator 3D spiral sequence showing one RR interval. The adiabatic inversion pulse is played out at delay time (TD) such that imaging is performed in the diastolic rest period. A navigator restore (selective inversion pulse) immediately follows to reinvert signal for the navigator, which is performed prior to a spatial saturation band, positioned over the chest wall and the chemical shift saturation pulse for fat signal. The imaging block is played out such that the acquisition of kz = 0 is at the inversion time TI. All 8 kz encoding steps for one of 16 spiral interleaves are acquired on alternate RR intervals with data acquisition window of 102.4 msec.

Free-breathing 3D spiral imaging was performed using a navigator-gated inversion prepared stack of spirals (TE = 0.6 msec, TR = 12.8 msec, flip angle = 20°). Sixteen spiral interleaves were required to fill kx–ky space with a field of view of 350 × 350 mm and an acquired spatial resolution of 1.4 × 1.4 mm (reconstruction to 0.7 × 0.7 mm with Fourier interpolation). In order to minimize off-resonance blurring, the duration of the spiral readout was short (9.6 msec). In addition, second-order gradient shimming and center frequency adjustments were performed up to three times on a user-defined cuboid volume positioned over the LV region. The slab thickness was 80 mm with 8 kz steps resulting in 8 slices of 10 mm reconstructed to 16 slices of 5 mm with Fourier interpolation. In general, the first image was discarded due to through-plane wrap resulting from an imperfect slab profile. All kz steps for a given interleaf were acquired in a single cardiac cycle (Fig. 1), resulting in an acquisition window of 102.4 msec. Respiratory gating was performed using a crossed-pairs navigator positioned on the dome of the right hemidiaphragm with a navigator acceptance window of 5 mm. For one patient the navigator was repositioned over the dome of the left hemidiaphragm as the right hemidiaphragm trace had an artifact superimposed on the lung/liver interface. A navigator-restore pulse 26 was implemented immediately after the inversion preparation to ensure adequate SNR in the navigator trace. End expiratory tracking with adaptive position of the acceptance window 27 was used in order to ensure that respiratory drift did not result in excessively poor navigator efficiency. Data were acquired on alternate cardiac cycles with acquisition duration of 33 cardiac cycles, including one dummy double cardiac cycle (assuming 100% navigator efficiency). A chest wall saturation band was applied prior to chemical-shift fat saturation, slab excitations, and spiral readouts to minimize signal from residual chest wall motion. The inversion time was estimated from that of the last conventional 2D breath-hold image performed. Spiral data were acquired with an anterior 18 element (3 rows of 6) body matrix coil and 2, 4-element, spine coils. During online reconstruction the coil elements furthest from the heart were automatically discarded (two from the spine coils and three from each row of the body matrix coil) to reduce image wrap. Raw data were exported to allow a global off-resonance correction where necessary. Images were reconstructed with a range of central frequency offsets 28 and the frequency giving the sharpest reconstruction over the LV determined.

### Image Analysis

Basal, mid, and apical slices with 30 mm separation were selected from the breath-hold 2D Cartesian images and matched with the closest respective images from the free-breathing 3D spiral acquisition. Images were zoomed to display the LV only and separated into basal, mid, and apical groups. Within each group, the image order was randomized. Two blinded assessors, each with >20 years of MR experience, scored the images on three parameters: overall image quality on a scale of 1 to 5 (1 = nondiagnostic to 5 = excellent), ghosting artifact presence on a scale of 0 to 2 (0 = none, 1 = moderate, 2 = severe); image sharpness on a scale of 1 to 3 (1 = poor to 3 = good). The assessors scored the images independently and in cases where they differed reached a consensus score. A paired Wilcoxon signed rank sum test was used to assess the significance of differences in scores between the sequences. Data were analyzed for each slice group (basal, mid, apical) and for all slices combined.

Additionally, the sharpness of the LV blood pool / myocardium interface was examined quantitatively using MATLAB (MathWorks, Natick, MA). First the endocardium border was defined using smoothed regions of interest (ROIs) on both the 2D Cartesian and corresponding 3D spiral images. For each of the basal, mid, and apical 2D slices, line profiles (3 pixels wide) normal to the endocardium border were obtained at three user-selected points (anteroseptal, septal, and inferoseptal). For each line profile, sharpness of the endocardial border was calculated as the inverse of the distance between 0.8 and 0.2 of the difference of the maximum and minimum pixel intensity 29. Sharpness was also calculated at corresponding locations on equivalent slices of the 3D spiral datasets. Differences between sequences were again assessed using paired Wilcoxon signed rank sum tests.

For each slice, ROIs were defined in the LV blood pool, ensuring to avoid trebeculae and valves, and in the myocardium. Blood SNR was defined as the ratio of mean blood pool intensity divided by the standard deviation of signal in a region of free space outside the chest wall, avoiding any major wrap or chest wall ghosting artifacts on both sets of images. Myocardium/blood CNR was defined as the difference in signal intensity between blood and myocardium divided by the standard deviation of the intensity in the region of free space. Normality tests showed that blood SNR and myocardium/blood CNR values were not normally distributed. Consequently, differences between sequences were assessed using paired Wilcoxon signed rank sum tests. Data were analyzed for each slice group (basal, mid, apical) and for all slices combined. For all statistical tests *P* < 0.05 was considered significant.

For all patients with focal regions of enhancement, the enhancement ratio between enhanced regions and nulled myocardium was measured and also the volume of the enhancement for the breath-hold 2D Cartesian images and free-breathing 3D spiral images. Images were analyzed using MATLAB. The endocardium and epicardium were defined using smoothed ROIs and a region of nulled myocardium selected. Enhanced tissue was then defined as all pixels between the endocardium and epicardium ROIs with intensity greater than 2 SDs above the mean of selected nulled tissue 1. The enhancement ratio was calculated as (S_enh_ – S_null_)/S_null_ where S_enh_ is the mean signal intensity in the enhanced region(s) and S_null_ is the mean signal intensity in nulled regions 25. Enhancement volume was calculated as the sum of enhanced pixels multiplied by the spatial resolution, using slice thickness of 10 mm (7 mm slice + 3 mm slice gap) for 2D Cartesian images and 5 mm for 3D spiral images.

## RESULTS

From the 18 patients scanned, three patients were excluded from analysis due to arrhythmic periods (ectopic beats) during either or both of the breath-hold 2D or 3D spiral imaging. All the results and analysis presented are from the remaining 15 patients. The mean duration required to acquire the eight breath-hold 2D slices covering the same volume as the 3D spiral acquisition, including rest periods between scans, was 230 ± 65 seconds (range: 116–311 sec). The free-breathing 3D spiral scans had a mean duration significantly (*P* < 0.001) shorter, 85 ± 28 seconds (range: 45–136 sec). The mean respiratory efficiency for the 3D spiral acquisitions was 45 ± 14% (range: 26–69%). For all patients the 3D spiral scan was completed in a shorter time than the equivalent stack of eight 2D breath-hold images.

After initial visual inspection, off-resonance blurring was apparent over the LV in 5 of the 15 patients; for these a global off-resonance correction was performed offline to improve image quality prior to analysis.

**Figure 2:**
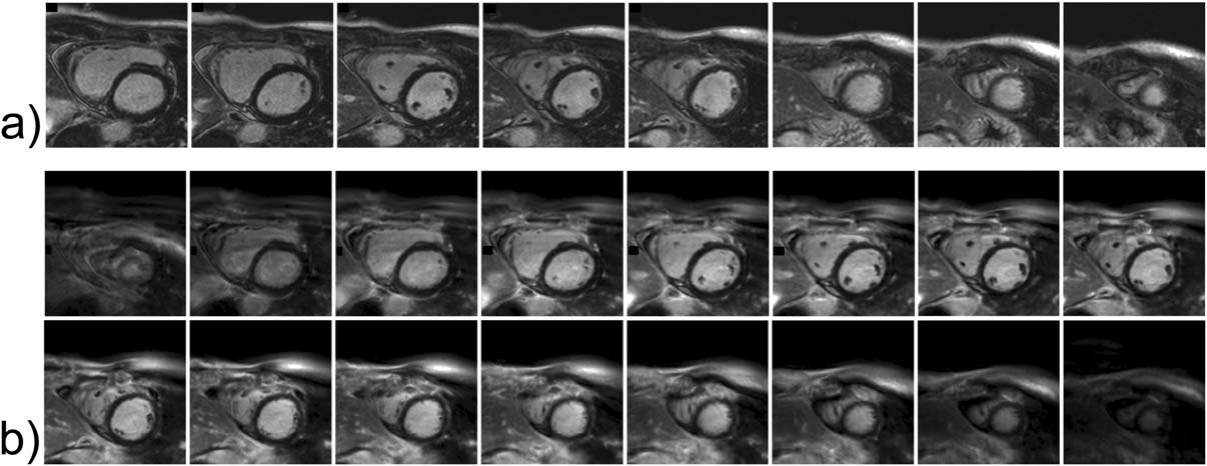
Example LGE images from one patient showing the LV short-axis stack from (a) the conventional breath-hold 2D Cartesian sequence and (b) the equivalent free-breathing 3D spiral volume images (16 reconstructed slices).

**Figure 3:**
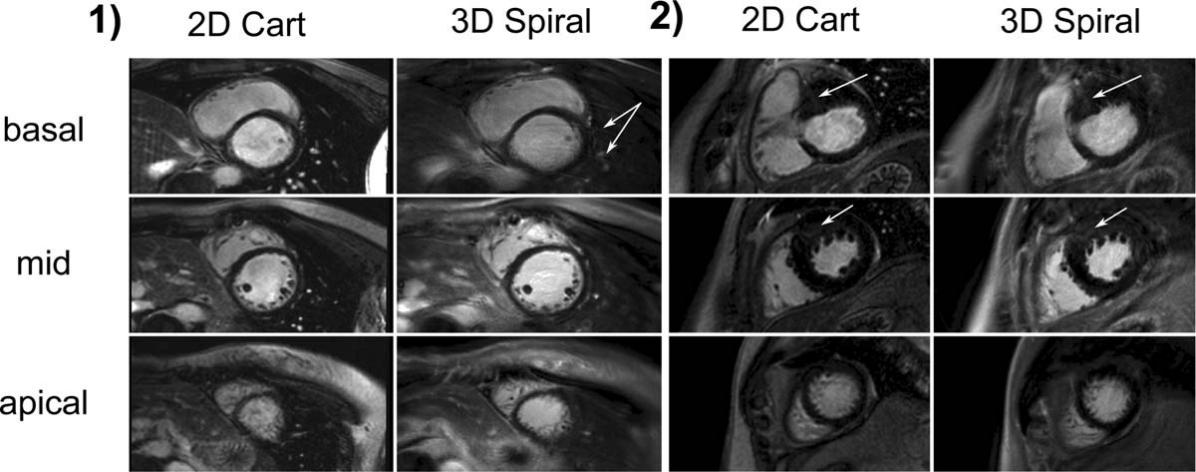
Example LGE images from two patients showing the three LV short-axis slices (basal, mid, and apical) used for visual scoring and SNR and CNR measurements. For each patient the left column shows conventional breath-hold 2D Cartesian images and the right shows the equivalent matched free-breathing 3D spiral images. Patient 1 has no visible enhancement but shows improved definition on the 3D spiral images, most visible on the trebeculae of the mid slice, a low degree of chest wall ghosting is present on the 3D spiral basal slice (arrowed). Patient 2 has patchy enhancement in an anterior hypertrophic region on both basal and mid slices (arrowed).

Example images from a subject showing eight breath-hold 2D slices and the corresponding 3D spiral images are shown in Fig. 2. Fig. 3 shows the basal, mid, and apical 2D Cartesian and 3D spiral images used for both visual scoring and also SNR and CNR measurements in two example subjects. In the first subject there is no late enhancement, while in the second there is diffuse/patchy enhancement within a hypertrophic region in both the basal and mid slices.

The mean consensus qualitative scores are summarized in Table1. For both 2D Cartesian and 3D spiral data, image quality was highest in the mid-slice and lowest in the apical slice. For the basal slice group, the image quality scores were higher for the 2D Cartesian acquisitions than for the 3D spiral acquisitions (3.9 ± 0.5 vs. 3.2 ± 0.6, *P* = 0.02). The artifact scores were also significantly lower for the 2D Cartesian acquisitions (0.07 ± 0.26 vs. 0.13 ± 0.26, *P* = 0.04) in this slice group. Image sharpness scores were equivalent. For the mid and apical slice groups, there were no significant differences in image quality, artifact scores, or sharpness scores between the two sequences. When looking at the combined slice groups, the image quality of the 2D Cartesian images showed a small but significant increase over that of the 3D spiral data (3.7 ± 0.9 vs. 3.4 ± 1.1, *P* = 0.03).

**Table 1:** Qualitative Scores for Breath-Hold 2D Cartesian and Free-Breathing 3D Spiral Images in the Basal, Mid, and Apical Slice Groups

	2D BH Cartesian	FB 3D Spiral	*P-*value
Basal			
Image quality	3.87 (0.52)	3.20 (0.56)	0.02^*^
Artifact	0.07 (0.26)	0.13 (0.26)	0.04^*^
Sharpness	2.13 (0.52)	2.00 (0.53)	0.73
Mid			
Image quality	4.33 (0.62)	4.07 (1.03)	0.40
Artifact	0.13 (0.35)	0.33 (0.62)	0.38
Sharpness	2.33 (0.49)	2.20 (0.68)	0.69
Apical			
Image quality	2.87 (0.74)	2.80 (1.08)	1.00
Artifact	0.27 (0.46)	0.33 (0.62)	1.00
Sharpness	1.27 (0.46)	1.60 (0.74)	0.19
Combined			
Image quality	3.69 (0.87)	3.36 (1.05)	0.03^*^
Artifact	0.16 (0.37)	0.40 (0.58)	0.16
Sharpness	1.91 (0.67)	1.93 (0.69)	0.83

Images were scored for image quality (1 = nondiagnostic to 5 excellent), the presence of ghosting artifact (0 = none, 1 = moderate, 2 = severe), and image sharpness (1 = poor to 3 = good). Values are reported as mean (± SD), together with the *P*-value from a Wilcoxon paired signed rank test. Results are also presented for the basal, mid, and apical slice groups combined (^*^P < 0.05).

Quantitative image sharpness measures are shown in Table2 for each slice group and all slices combined. No significant differences were found between the sharpness measures.

**Table 2:** Quantitative Results for Measurement of Image Sharpness for Breath-Hold 2D Cartesian and Free-Breathing 3D Spiral Images in the Basal, Mid, and Apical Slice Groups

	BH 2D Cartesian (mm^−1^)	FB 3D Spiral (mm^−1^)	*P-*value
Basal	0.426 (0.075)	0.444 (0.110)	0.56
Mid	0.416 (0.057)	0.385 (0.065)	0.22
Apical	0.400 (0.066)	0.362 (0.075)	0.12
Combined	0.414 (0.066)	0.397 (0.090)	0.25

Values are reported as the mean sharpness of three selected points around the endocardial border with the LV blood pool from all patients for each of the individual slice groups. Results are also presented for all slices combined.

Table3 shows the mean LV blood pool SNR and LV blood pool/myocardium CNR for the basal, mid, and apical slice groups and for all slice groups combined. There were no significant differences in the SNR and CNR values for the 2D Cartesian and 3D spiral acquisitions.

**Table 3:** Quantitative Results From Measurements of LV Blood Pool SNR and LV Blood / Myocardium CNR for Breath-Hold 2D Cartesian and Free-Breathing 3D Spiral Images in the Basal, Mid, and Apical Slice Groups

	BH 2D Cartesian	FB 3D Spiral	*P-*value
Basal			
LV blood SNR	135.6 (55.3)	152.1 (88.7)	0.39
Blood/Myo CNR	114.4 (46.8)	115.5 (71.1)	0.85
Mid			
LV blood SNR	127.0 (57.2)	155.1 (71.1)	0.09
Blood/Myo CNR	110.0 (50.9)	121.3 (57.0)	0.39
Apical			
LV blood SNR	102.0 (42.9)	102.1 (65.2)	0.64
Blood/Myo CNR	80.3 (42.9)	71.5 (46.1)	0.21
Combined			
LV blood SNR	121.5 (55.3)	136.4 (77.9)	0.13
Blood/Myo CNR	101.6 (48.4)	102.7 (61.8)	0.98

Values are reported as mean (± SD), together with the *P*-value from a Wilcoxon paired signed rank test. Results are also presented for the basal, mid, and apical slice groups combined (^*^P < 0.05).

Of the 15 patients analyzed, five showed regions of late enhancement. All regions of enhancement were visualized on both breath-hold 2D Cartesian and free-breathing 3D spiral images. Two of the five patients had patchy, diffuse enhancement within hypertrophy regions and were not analyzed further. The remaining three patients had focal regions of enhancement on 5, 2, and 2 2D Cartesian images and on 9, 3, and 4 3D spiral images, respectively. The mean enhancement ratios and total enhanced volumes are shown in Table4. Averaged over all three patients, the enhancement ratios were 0.65 and 0.63 and the enhanced volumes were 13.8 cm^3^ and 14.1 cm^3^ for the 2D Cartesian and 3D spiral images, respectively.

**Table 4:** Measurements of the Enhancement Ratio and the Volume of Enhanced Regions

Patient (No. of Slices: 2D / 3D)	Enhancement Contrast Ratio		Enhanced Volume (cm^3^)	
	BH 2D Cartesian	FB 3D Spiral	BH 2D Cartesian	FB 3D Spiral
1 (5/9)	0.65	0.69	10.86	14.12
2 (2/3)	0.65	0.60	16.64	17.35
3 (2/4)	0.65	0.58	13.91	10.89

Defined using automatic segmentation (2 SD above the mean of manually defined region(s) of normal, nulled myocardium for patients with regions of focal enhancement. Also shown is the number of slices on which enhancement was present for both the 2D Cartesian and 3D spiral scans.

## DISCUSSION

We have demonstrated the use of a navigator gated 3D stack of spirals LGE imaging sequence to acquire the LV short axis stack in a statistically significant and substantially reduced acquisition time. On average, the acquisition of the free-breathing 3D spiral data was 3.7 times faster than the multiple breath-hold 2D Cartesian acquisitions covering the same volume.

Qualitative image quality scores from the two sequences were similar, although for the basal slice they were significantly higher for the 2D Cartesian images. Qualitative sharpness scores were also slightly but significantly better for the 2D Cartesian sequence in the basal slice, although this was not the case for quantitative assessment. While the shorter acquisition window of the 3D spiral sequence should reduce motion effects and therefore improve image quality, in practice this will be very dependent on the *k*-space acquisition order. In this study, all of the through-plane phase encoding steps for a given spiral interleaf were acquired in each cardiac cycle so, although the acquisition window was reduced relative to the Cartesian acquisition, any motion within that window would be deleterious and could result in a slightly reduced image quality relative to the Cartesian studies. Future work may include investigating the effect of *k*-space ordering on image quality.

Off-resonance blurring was often present in the RV free wall on the 3D spiral images. It was also sometimes present in the region of the inferior myocardium close to the lung and spleen interface next to the mid to apical LV inferior wall. Both of the previous studies that used spiral readouts for 3D LGE imaging were performed at 1.5T, where off-resonance is less of a problem 23,25. In this study, the combination of a short spiral read duration and careful shimming minimized off-resonance blurring and achieved similar image sharpness compared to the breath-hold 2D images. A global off-resonance correction was applied offline to improve image quality over the LV for five of the patients. The use of a spatially varying off-resonance correction, as used by both Knowles et al 23 and Shin et al 25, would further reduce blurring and improve image quality. However, access to the field maps required to implement this was not available in this study. Spatially varying off-resonance correction may also allow an increase in spiral readout duration, which could be used in turn to reduce the number of required interleaves and scan duration.

Image artifact scores were slightly and significantly higher for the spiral data at the basal level than for the Cartesian data. The main source of artifact in the 3D spiral acquisitions was ghosting of the chest wall due to residual respiratory motion, which was seen in some 3D spiral studies despite using a chest wall saturation band. In addition, despite having a much reduced acquisition window, the acquisition of all through-plane phase encoding steps for a given spiral interleaf within each cardiac cycle may exacerbate sensitivity to residual respiratory motion, as discussed above. One solution would be to acquire all of the data within a single breath-hold: using single RR interval gating with the current 3D spiral parameters would allow full data acquisition in 17 cardiac cycles (including one dummy cycle). While this is shorter than the majority of the breath-hold 3D LGE techniques currently reported 6,8–10, it is too long for many patients. The use of parallel imaging would allow the acquisition to be reduced to more acceptable breath-hold durations and this has recently been reported at 1.5T by Shin et al 25 using SPIRiT 24. At 3T, however, the longer T_1_ of normal myocardium would make a single R-wave gated acquisition more sensitive to heart rate variations. In fact, even with the alternate R-wave gating used in this study, the increased T_1_ at 3T would result in increased sensitivity to RR interval variability. In our study, this would always be worse for the 3D spiral acquisition, as it was always performed after the Cartesian study (so as not to compromise the clinical examination) when T_1_s were longer.

Despite all free-breathing 3D spiral imaging being performed after the final 2D Cartesian imaging and starting ∼20 minutes post contrast injection, the quantitative measures of LV blood pool SNR and blood/myocardium CNR were similar for the free-breathing 3D spiral and the breath-hold 2D images. Consistently acquiring the 3D spirals scans second, when the T_1_ of the myocardium is longer due to increased gadolinium washout, should result in reduced SNR and CNR. It also potentially results in increased sensitivity to RR interval variations, although this is mitigated by using alternate R-wave gating. A future study with random ordering of the acquisitions would avoid any bias.

**Figure 4:**
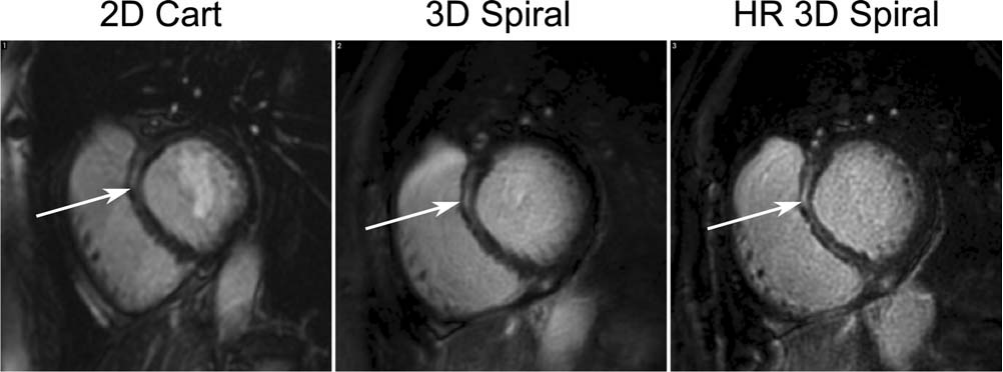
Images of a basal slice from a patient showing enhancement in the mid-wall of the septum (arrowed). From left to right the images are from the breath-hold 2D (7-mm slice thickness), free-breathing 3D spiral (5-mm reconstructed slice thickness), and a higher through-plane free-breathing 3D spiral (1.5-mm reconstructed slice thickness) centered on the enhancement region. The higher-resolution scan shows better definition of the enhanced region in the mid-wall.

Of the 15 patients analyzed, only three exhibited focal regions of enhancement, a limitation of this study. The enhancement ratio was found to be similar for all of the patients. The improved resolution of the 3D spiral sequence should allow better depiction of the scar region, especially in cases where misregistration of nominally contiguous 2D slices occurs. The further example in Fig. 4 shows the increased detail that can be seen with acquired higher through-plane resolution.

The imperfect profile of the slab excitation resulted in through-plane wrap, which was only obviously present on one image at the end the stack (Fig. 2). To obtain full coverage of the LV requires a usable image volume of length 80–120 mm (patient-dependent), requiring 8–12 kz encoding steps. While maintaining slice resolution, this would extend the acquisition window to 128 and 154 msec for 10 or 12 kz steps, respectively, which remain at the lower end of the breath-hold 2D Cartesian acquisition window range (∼130–210 msec) but increases the potential for cardiac motion blurring. Partial Fourier is a method that was employed by many of the previous breath-hold 3D Cartesian scans and would improve through-plane coverage without the need for acquiring extra kz partitions and the associated increase of the acquisition window and is currently being investigated.

Due to the short scan duration, on average 85 seconds for the 3D spiral volume, multiple repetitions of the free-breathing 3D spiral scans could be performed in the same duration as required to perform the breath-hold 2D stack. Multiple repetitions could also allow the acquisition of alternate views such as a long axis stack or the acquisition of higher-resolution scans of any regions showing enhancement. This is demonstrated in Fig. 4, which shows basal images from an additional subject not included within the study with a well-defined region of enhancement in the septum from three different acquisitions; 1) breath-hold 2D Cartesian, 2) free-breathing 3D spiral (16 × 5 mm), and 3) high-resolution free-breathing 3D spiral (16 × 1.5 mm). The 3D spiral image better defines the enhancement region due to the higher in-plane and through-plane resolution. Further examination of the higher through-plane resolution 3D spiral scan shows even greater definition of the enhancement region.

In conclusion, navigator-gated 3D spiral scans may offer an alternative to conventional 2D breath-hold scans for viability assessment of the left ventricle, particularly for patients who may struggle to breath-hold. The 3D spiral scans had much reduced acquisition durations that allow multiple acquisitions in different planes or with higher resolution within the same duration of a standard 2D breath-hold stack. Although the 3D spiral scans had a reduction in image quality, they had similar SNR and CNR, while providing higher through-plane resolution compared to the 2D Cartesian scans.
